# Patient-Derived Spheroid Culture Models Are Better Than Monolayer Models in Chondrosarcoma Research

**DOI:** 10.21203/rs.3.rs-3728259/v1

**Published:** 2023-12-13

**Authors:** Ruichen Ma, Tanya Heim, Karen Schoedel, Kurt R. Weiss

**Affiliations:** China-Japan Friendship Hospital; University of Pittsburgh School of Medicine; University of Pittsburgh; University of Pittsburgh School of Medicine

**Keywords:** Chondrosarcoma, spheroids, cytotoxicity, treatment

## Abstract

**Purpose:**

Chondrosarcoma (CSA) are mesenchymal tissue-derived bone tumors. CSA mainly occurs in older people. CSA has demonstrated resistance to chemotherapy and radiation; complete surgical removal with negative margins is the only treatment option. In the case of metastatic CSA, the chance of survival is meager. Since the conventional two-dimensional cell culture models failed to retain tumor characteristics, developing preclinical models mimicking the disease with the highest fidelity is paramount for personalized treatments.

**Methods:**

In this study, we established spherical cultured cells as new models for CSA. First, we demonstrated that CSA cells could form spheroids when cultured in ultra-low attachment plates. Next, tissue samples from CSA patients were collected and processed into primary cells, which were subsequently cultured as primary spheroids. The growth rate of primary spheroids was monitored and the histology of mature spheroids were characterized. These primary spheroids were used in drug susceptibility studies where traditional doxorubicin therapy and our novel disulfiram-copper therapy were tested.

**Results:**

Compared with conventional monolayer cultures, spheroids better recapitulated the features of the in vivo tumor in the aspect of the formation of extracellular matrix. In the drug susceptibility study, spheroids demonstrated high resistance to the classic therapies, suggesting that monolayer cultures may give false positive results. Therefore, using spheroids for drug research and development in the CSA field should provide more accurate results.

**Conclusion:**

In summary, our study of primary CSA spheroids brought new insight into their chemoresistance and demonstrated its potential for personalized treatment of CSA in clinical medicine.

## Introduction

Chondrosarcoma (CSA) is a collective term of a group of heterogeneous malignant bone tumors which are normally characterized by common production of hyaline cartilaginous matrix. CSA arise mostly in adults and is the second most common bone malignancy after osteosarcoma, accounting for approximately 20% of all bone malignancies, and it has been reported about 3 new cases per million each year ^1^. CSA is classified on a continuum of histologic grades, from low grade CSA to dedifferentiated CSA, and the incidence of metastases and prognosis are related to tumor grade ^2–4^. CSA is a so-called surgical disease, which means until now complete surgical removal of the lesion before metastases is the only way to cure ^5–7^. Therefore, it is necessary to furtherly understand the mechanism of CSA development and to develop novel treatment for those unresectable cases. However, one of the greatest barriers to CSA basic research is the lack of reliable models. CSA are usually abundant in extracellular matrix (ECM), and the conventional monolayer cultures fail to retain the complexities of tumor microenvironment of CSA ^8,9^. This lead to the development of three dimensional cultures, including spheroids, organoids and scaffold-based tissues ^10–12^. Among these options, spheroids are the most straightforward, and the protocol of culturing is easily standardized. Several studies have demonstrated the feasibility of CSA spheroids culture and advantages of the model in aspect of recapitulating tumor oxygen gradients, ECM formation and high resistance in cytotoxicity assay ^13–15^. However, whether there is difference between low grade CSA spheroids and dedifferentiated CSA spheroids remain to be investigated. We believe this would help to understand both CSA itself and the spheroids models.

In this study, we asked: (1) Are there differences between low grade and dedifferentiated CSA spheroids in aspects of morphology, histology and chemoresistance? (2) Do both types of patient derived CSA spheroids recapitulated tumor morphology and chemoresistance? We hypothesize that both low grade and dedifferentiated patient derived spheroids retain the own characteristics of their original tumors and thus, patient derived spheroids are ideal models for CSA basic research.

## Methods

### Patient samples and primary cell culture

Institutional review board approval (PRO10050461) was obtained from UPMC Shadyside Hospital for this study. Informed consent to participate in our tumor registry and tissue bank were obtained to collect samples from patients. Once the sarcoma has been removed from the operative field, the surgeon performs the intralesional removal of sarcoma tissue and the tumor was transported to the lab. Tumors were minced and dissociated into single-cell suspensions using the MAC human tissue dissociation kit (Miltenyi Biotec, Auburn, CA, USA). Primary cells were cultured in flasks (37° C, 5% CO2) with DMEM (Corning, Manassas, VA, USA). Medium was changed every three days or when medium turned yellow. Cells were harvested at 90% confluence and divided into two groups: 1) cryopreserving: P0 (Passage 0) in freezing medium containing 70% Dulbecco’s modified eagle medium, 20% fetal bovine serum, and 10% dimethyl sulfoxide (4-X-5, ATCC, Manassas, VA, USA) and 2) splitting into flasks for spheroid culturing as P1 (Passage 1).

### Spheroids culture

HT1080 (ATCC, CCL-121, Manassas, VA, USA), SW1353 (ATCC, HTB-94, Manassas, VA, USA) cell lines and P1 cells were seeded into Nunclon Sphera 96-well round bottom Microplates (Thermo Scientific, Rochester, NY, USA) with an initial density of 10,000–20,000 cells with 100 μL of medium per well (day 0). Another 100 μL of Fresh medium was added to each well after 3 days of seeding (day 3). The medium was changed every 3 days or when medium turned yellow. To change the medium, 100 μL of supernatant was gently aspirated from each well and 100 μL fresh medium was added. All the cultures were maintained in a 5% CO_2_ humidified environment at 37°C. Spheroid growth was monitored and images were captured using an Olympus IX81-Motorized Inverted Microscope (Olympus, Center Valley, PA, USA). Spheroid size was measured by cellSens Standard software (Olympus Life Science, Waltham, MA, USA).

### Agarose embedding and histology

Spheroids were cultured for 10 days, and medium was gently aspirated from each well. Spheroids were washed three times with PBS. Spheroids were fixed with 4% paraformaldehyde (Electron Microscopy Sciences, Hatfield, PA, USA) for 30 minutes. After fixation, spheroids were transferred to tissue culturing dishes and embedded with 4% agarose. Solidified agarose was cut into small cubes with spheroid in the center. Cubes were dehydrated successively in 70% isopropanol for 2 hours, 96% isopropanol for 2 hours, 100% isopropanol overnight and 100% acetone for 2 hours. Dehydrated agarose cubes were incubated in pre-warmed liquid paraffin for 2 hours before paraffin embedding. 5-μm-thick sections were cut and adhered to poly-_L_-lysine-coated glass slides. For monolayer culture, single cell suspension was obtained from flask after trypsinization and were seeded into Nunc Lab-Tek II Chamber Slide (Thermo Scientific, Rochester, NY, USA) at an initial density of 80,000 cells with 2 mL medium per chamber. After adherence to the slides, chambers were removed. Both monolayer culture and spheroids slides were stained with hematoxylin and eosin after deparaffinization and rehydration. Imaging was performed with the Nikon Eclipse E800 microscope (Nikon Instruments, Melville, NY, USA) at magnifications of 20 × and 40 ×. Images of the tumors; pathologic sections were taken with an Olympus BX45 microscope (Olympus, Center Valley, PA, USA) by a musculoskeletal pathologist in UPMC Shadyside Hospital.

### Cytotoxicity assay

For monolayer cultures, 20,000 cells/well were seeded into flat bottom 96-well plates (Corning Inc, Kennebunk, ME, USA) with 100 μL of culture medium and left to adhere overnight. Doxorubicin hydrochloride (LC Laboratories, Woburn, MA, USA), disulfiram (Sigma-Aldrich, St. Louis, MO, USA) were then added to each well. For 3D culture group, spheroids were seeded in Nunclon Sphera 96-well round bottom Microplates at density of 20,000 cells per well and were maintained for 7 days. Drugs were applied at day 8 of spheroids culture. Doxorubicin was diluted using DMEM, and three-fold dilution from the highest concentration of 100 μM to lowest concentration of 5 nM was achieved. Cells and spheroids were incubated in different concentrations of doxorubicin for 48 hours in a 5% CO2 humidified environment at 37° C, and the cell viability was measured. To measure cell viability after drug treatment, 10 μL of PrestoBlue (Invitrogen, Carlsbad, CA, USA) was added to each well and plates were placed in the incubator for 25 minutes for 2D cultures and 3 hours for spheroids. Fluorescence was measured with a Synergy HT Multi-Mode Microplate Reader (BioTek, Winooski, VT, USA) with an excitation and emission wavelength of 560/590 nm.

### Statistical analysis

All quantitative data in this study was generated from at least three independent biological replicates. All graphs are presented in form of mean value with standard deviation. Statistical analysis was performed using Graphpad Prism 8 (GraphPad, La Jolla, CA, USA). *p < 0.05, **p < 0.005, ***p < 0.001, ****p < 0.0001.

## Results

### Generating and Growth Monitoring of Low Grade CSA Spheroids

**Figure 1:**
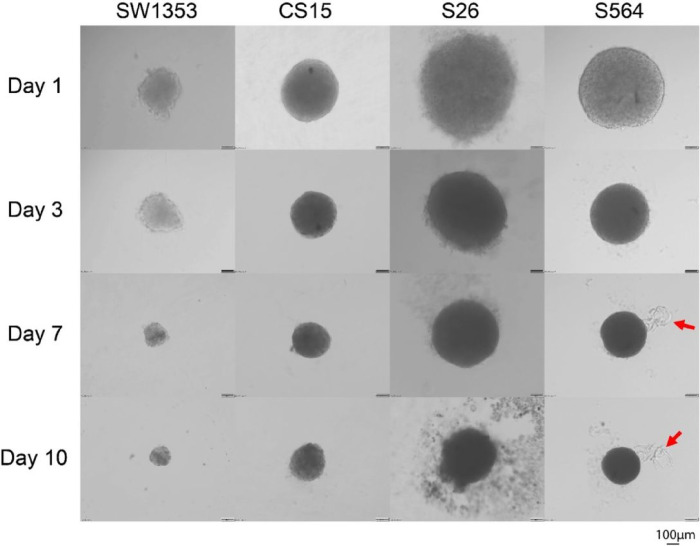
Growth of low grade CSA spheroids. Cell line SW1353 and three patient-derived primary cells CS15, S26, S564 were seeded into ULA plates at initial density of 20,000 cells per well. The formed spheroids were representatively imaged at time point of day 1, day 3, day 7 and day 10 respectively. For each sample, n = 10, scale bar = 100 μm. Red arrows indicates the transparent matrix secreted by S564 spheroid.

The spheroids generated from both cell line and low grade CSA patient samples could be maintained for over 10 days. After SW1353 was first seeded into ULA plates and incubated overnight, the SW1353 spheroid formed at day 1, with diameter of 354.17 ± 6.03 μm. The size of spheroids decreased from day 1 to day 7 (166.67 ± 5.44 μm). At day 10, the diameter was 158.33 ± 3.75 μm. For CS15, spheroids formed at day 1 with diameter of 483.33 ± 7.47 μm. After formation, spheroid shrunk and became denser in the core area. The diameter of day 10 spheroid was at 279.17 ± 5.62 μm. S564 cells grew in the same pattern as CS15 did. The diameter of day 1 S564 spheroid was at 688.24 ± 9.42 μm, and it decreased to 300.00 ± 5.81 μm at day 10. As red arrow shows in Fig. 1, we observed transparent matrix around S564 spheroid at day 7 and day 10, which did not exist at earlier stage (Fig. 1). The S26 spheroids were observed with the largest diameter of 770.83 ± 10.21 μm, compared with that of other samples at day 1. We observed the same growth pattern of S26 as that of other samples. The well-formed spherical shape was observed at day 7, and the diameter was of 508.33 ± 4.77 μm. At day 10 of culture, the S26 spheroids began to collapse and the main body of the spheroids were surrounded by lots of cell debris (Fig. 1).

### Generating and Growth Monitoring of Dedifferentiated CSA Spheroids

**Figure 2:**
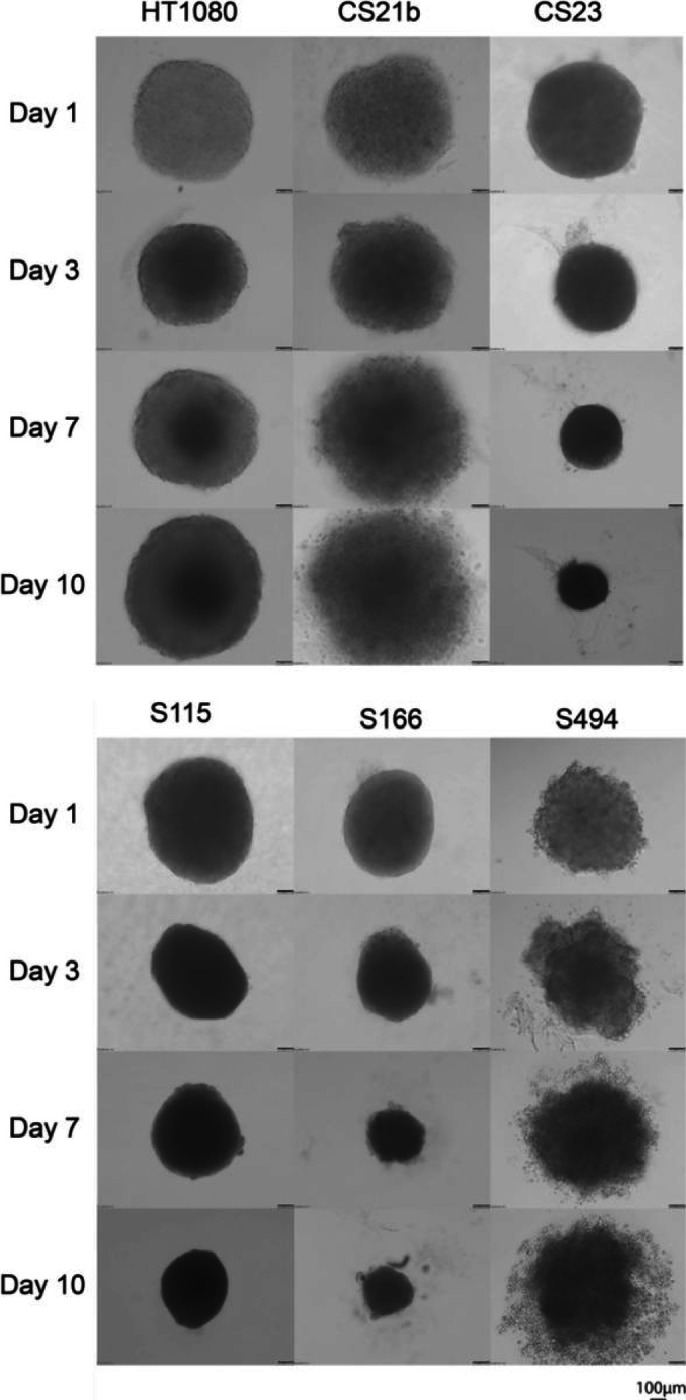
Growth of dedifferentiated CSA spheroids. CSA cell line HT1080, patient derived primary cells CS21b, CS23, S115, S166 and S494 were seeded into ULA plates at a density of 20,000 cells per well. The growth of spheroids were monitored at day 1, day 3, day 7 and day 10 under the microscope, at magnification of 10X. n = 10, scale bar =100 μm

HT1080 spheroids formed at day 1 with a diameter of 729.17 ± 10.56 μm, and increased in cell quantity but decreased in size (645.83 ± 6.27 μm) at day 3. The spheroid grew into a mature round shape and then proliferated and expanded from day 3. It reached diameter of 854.17 ± 11.77 μm at day 10, and the 2 layers structure that dark core and light peripheral area was observed under microscope. We observed two different growth patterns of dedifferentiated CSA spheroids. The first group included CS23, S115 and S166 spheroids which the shrunken growth patterns were observed. The size of day 1 CS23 spheroid was 708.33 ± 9.73 μm, and 729.17 ± 8.97 μm, 604.17 ± 7.52 μm for S115 and S166 respectively. At day 3, we observed the density of these 3 spheroids was significantly increased. From day 3 to day 10, the sizes kept decreasing and reached 301.67 ± 5.66 μm, 479.17 ± 4.43 μm, 301.67 ± 4.01 μm for CS23, S115, S166 correspondingly. At day 10, cell debris can be observed around S166 spheroids. The second group consisted of CS21b and S494. After formation of spheroids, CS21b and S494 spheroids enlarged from diameter of 750.00 ± 6.98 μm and 695.65 ± 8.84 μm at day 1, to diameter of 958.33 ± 14.62 μm and 739.13 ± 10.66 μm at day 10 correspondingly (Fig. 2).

### Size of Spheroids Differed from Low Grade CSA Group to Dedifferentiated CSA Group

**Figure 3:**
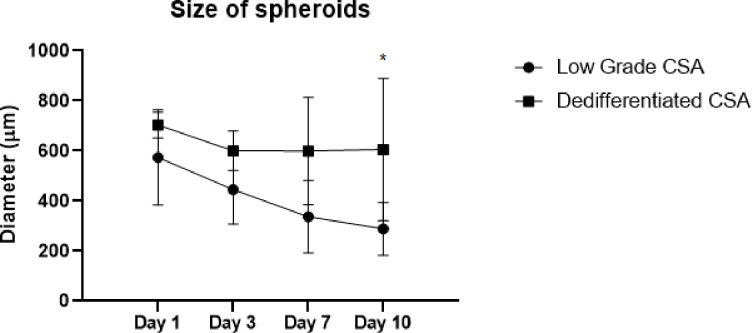
Size changes of two group CSA spheroids at different time points. The sizes of both low grade and dedifferentiated groups of spheroids were measured and plotted. N_low grade_ = 4, N_dedifferentiated_ = 6, *p < 0.05

Based on our bright field microscopy images of two pathological groups of low grade CSA and dedifferentiated CSA spheroids, we found the average size of dedifferentiated CSA spheroids were larger than that of low grade CSA spheroids, though we seeded the same amount of cells into each well. The significant difference (p = 0.029) in spheroid size (288.54 ± 91.64 μm v.s. 605.69 ± 91.64 μm) was observed in day 10 (Fig. 3).

### Histology of primary cells, spheroids and original low grade tumors

**Figure 4:**
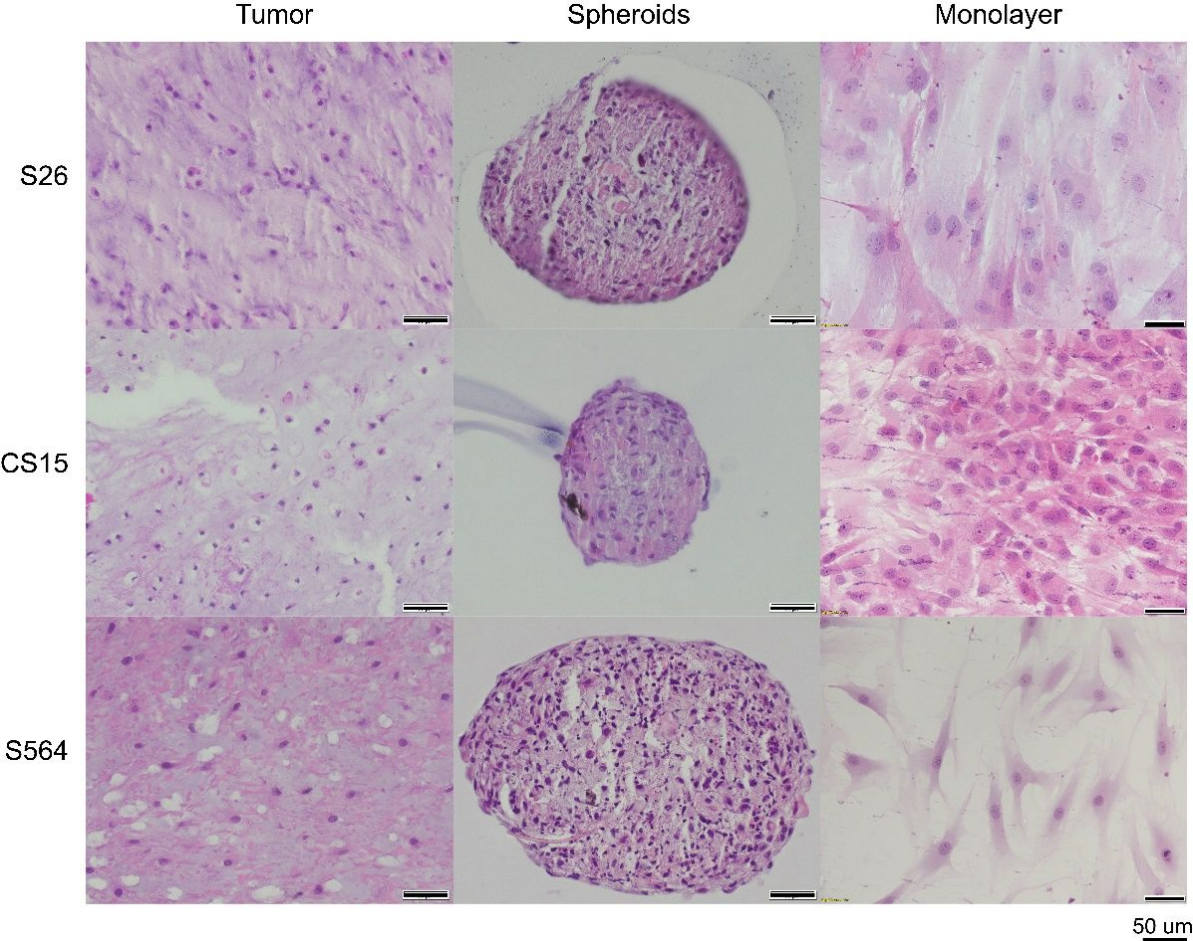
H&E Staining of Primary Cells, Spheroids and Low Grade Tumors. S26, CS15 and S564 were included. The primary cells formed spheroids and original tumors were stained with H&E. Images were taken at 40X magnification for tumors and spheroids, and at 20X for monolayers. Scale bar = 50 μm, n=6.

In the S26 tumor, abundant hyaline matrix was observed with an approximate cellularity at 40% – 60%. The nuclei of cells were round and occasionally spindled. Two mitosis per 10 high power field (hfp) was recorded. In S26 spheroid, some eosinophilic matrix was observed and the cellularity was scored between 40% –60%. Nuclei of cells were spindled and occasionally degenerated. Clear boundaries were formed by spindled cells at the periphery. The size of monolayer cells was large, and confluence was at 95%. For CS15, small, round oval cells were observed in the tumor and the cellularity was of 20% – 40%. Stellate and hyperchromatic nuclei were observed. The ECM was abundant. The corresponding spheroid was formed by pleomorphic spindle cells with cellularity at 40% – 60%. ECM was observed inside. Some cells were degenerated. The cytoplasm was granular in the spheroid. The boundary was well formed. The monolayer cells were at 100% confluence and mitoses were observed. In S564 tumor, vacuolated cells were observed. The cellularity of the tumor was at 40%–60%. Hyaline matrix was formed and the eosinophilic component could also be observed. In the spheroid, the cellularity was of 60% – 80%. Both spindled cells and round cells were observed and pleomorphic was presented. Vacuolation was found inside the spheroid. The boundary was well maintained by cells in periphery. For monolayer cells, they were lightly stained and were at confluence of 40% (Fig. 4).

### Histology of primary cells, spheroids and original dedifferentiated tumors

**Figure 5:**
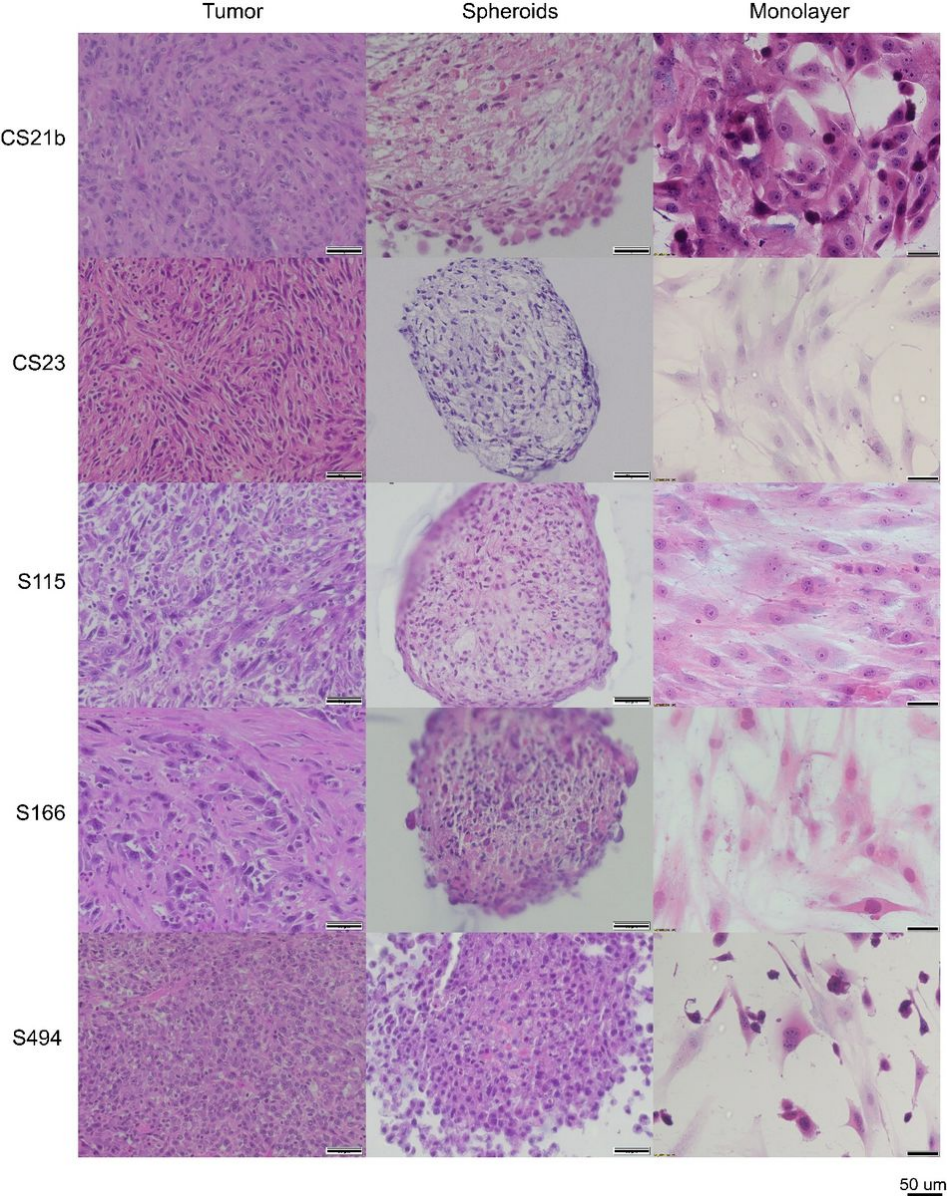
H&E Staining of Primary Cells, Spheroids and Original Dedifferentiated Tumors. CS21b, CS23, S115, S166 and S494 were included. The primary cells formed spheroids and original tumors were stained with H&E. Images were taken at 40X magnification for tumors and spheroids, and at 20X for monolayers. Scale bar = 50 μm, n=6.

Dedifferentiated CSA tumors demonstrated similarities that they all had high cellularity and presented low ECM formation. For CS21b, pleomorphic and spindle cells were observed in the tumor. Neutrophils and lymphocytes infiltrated. The mitotic activities were at 60 per 10 hpf. For corresponding spheroid staining, the density of cells was increased at the edge and decreased in the core area. In the middle of the spheroids, cells were observed more spindle shaped and degenerated cells could also be found. Round cells were observed mainly at peripheral area, and there were detached individual cells at the area. The CS21b 2D cells were at about 90% confluence, and the nuclei were deeply stained. Mitosis was observed in the monolayer cultures. For CS23, the tumor was of about 100% cellularity with a few lymphocytes infiltrating. Cells were pleomorphic and spindled. Mitotic activity was about 115 cells per 10 hpf. In spheroid, cellularity was at about 60% – 80%. Cells were vacuolated in the middle and degenerated cells were observed. Cells at the periphery were spindled and a clear capsule-like boundary formed. There were no cells detached from the spheroid. For monolayer cells, they were at 50% confluence and were lightly stained by H&E. For S115, there was no ECM formation in the tumor and cellularity was at 60% – 80%. Cells were pleomorphic and some rhabdoid cells presented. Lymphocyte infiltration was also observed. Mitosis was found about 15 cells per 10 hpf. ECM was not formed and cellularity was about 80% – 100%. Pleomorphic and vacuolated cells were located in the middle. The cells at the outer layer were deeply stained and were all spindled, therefore a capsule-like boundary formed, and no cell was detaching. S166 tumor was composed of pleomorphic oval cells. Both round and spindled nuclei were observed. The cytoplasm was amphophilic. Neutrophils and lymphocytes infiltrated. 26 mitotic cells were observed per 10 hpf. The cellularity of the tumor was about 60% – 80%. In the corresponding spheroid, most of cells were round and a few were spindled. Degenerated cells also existed in the spheroids. The S166 monolayer cells were imaged at about 60% confluence. In S494 tumor, lots of lymphocytes infiltrated and cells were observed in round shapes with pleomorphic nuclei. ECM was not found in the tumor and cellularity was about 80% – 100%. The mitotic activity was high as 70 cells per 10 hpf. In the spheroids, cells were at high density at the peripheral area compared with cells in the core. Whereas lots of cells were loosely attached and detached cells were also observed. The cellularity of the spheroid was about 80% – 100%. Cells were round with pleomorphic nuclei. The confluence of corresponding monolayer cells was about 30% (Fig. 5).

### Spheroids Recapitulate Tumors Better in Morphology Compared with Monolayer Cultures

**Figure 6:**
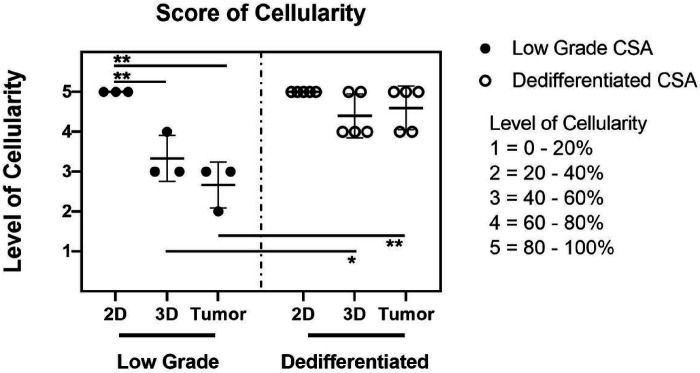
Scored cellularity of low grade CSA and dedifferentiated CSA. The cellularity of monolayer cultures (2D), spheroids (3D) and tumors were evaluated according to pathologic criterion. Cellularity were semi-quantitatively divided into five intervals, from 0–20% to 80–100% and scored correspondingly as level 1 to 5. The low grade and dedifferentiated CSA groups were scored separately. *p < 0.05, **P < 0.001.

We compared scores of cellularity of low grade tumors to that of dedifferentiated tumors. The mean value of the low grade group was 2.667 and the mean value of the dedifferentiated group was 4.600. Significant differences were demonstrated between the two groups of tumors (p = 0.003). Comparing spheroids between two different groups, the cellularity score of dedifferentiated spheroids (mean value = 4.400) was significantly (p = 0.040) higher than that of low grade spheroids (mean value = 3.333). we also compared scores of samples in the same group. The cellularity of monolayer cultures was at 100% in both groups. When comparing between monolayer cultures, spheroids and tumors in dedifferentiated groups, no significant difference was observed. In the low grade CSA group, we observed a significant decrease of score in the spheroids (p = 0.0088) and tumors (p = 0.0016) when compared to the monolayer cultures. Furthermore, there was no marked differences in cellularity between the low grade CSA spheroids and tumors (Fig. 6).

### Spheroids Have Higher Resistance towards Doxorubicin Treatment Compared with Monolayer Cultures

**Figure 7:**
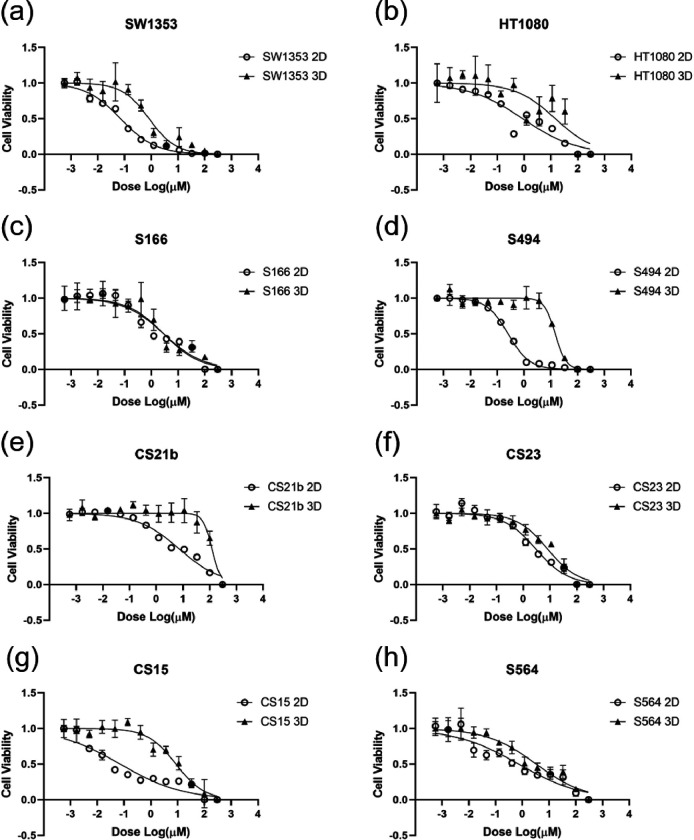
Doxorubicin treatment on 2D and 3D cultures. Two CSA cell line (a) SW1353, (b) HT1080, and six primary cells (c) S166, (d) S494, (e) CS21b, (f) CS23, (g) CS15 and (h) S564 were cultured as both monolayer (2D) and spheroids (3D). Cells were treated by doxorubicin at different dose. Data was fitted by nonlinear curve (four parameters) with least squares. Five independent cultures were treated and measured by PrestoBlue.

2 CSA cell lines (HT1080 and SW1353) and 6 CSA primary cells (S166, S494, CS15, CS21b, CS23, S564) were cultured as both monolayer cells and spheroids. Cultures were treated by different doses of doxorubicin. For SW1353, IC50 of 2D cultures were at 70.8 nM and IC50 of spheroids were at 0.827 μM (Fig. 7a). The IC50 of HT1080 monolayer cells and spheroids were 0.875 μM and 14.56 μM respectively (Fig. 7b). We next performed the assay on our patient-derived spheroid models. S166 was the only one that we did not observe significant differences between 2D and 3D cultures. The IC50 of S166 2D and 3D cultures was 2.54 μM and 3.26 μM respectively (Fig. 7c). Large differences were observed between S494 monolayer cells and spheroids, the IC50 of which was 0.255 μM and 14.7 μM correspondingly (Fig. 7d). In CS21b, significant differences in IC50 was also observed between two models, with 7.26 μM of monolayer cultures and 121.9 μM of spheroids (Fig. 7e). CS23 spheroids (IC50 = 8.09 μM) were more resistant to doxorubicin compared with monolayer cells (IC50 = 2.96 μM) (Fig. 7f). The same pattern was also observed in CS15 and S564 samples, the spheroids of which were more resistant to corresponding monolayer cultures. The IC50 of CS15 2D cells was 71.9 nM and of 3D cells was 8.70 μM (Fig. 7g). The IC50 of S564 monolayer cells was 0,723 μM and 0f spheroids was 3.30 μM (Fig. 7h). We summarized the R-square values of these curves, corresponding LogIC50, as well as p values of comparison of fits in Table 1.

### IC50 of the Culture Was Related to Several Factors

**Figure 8:**
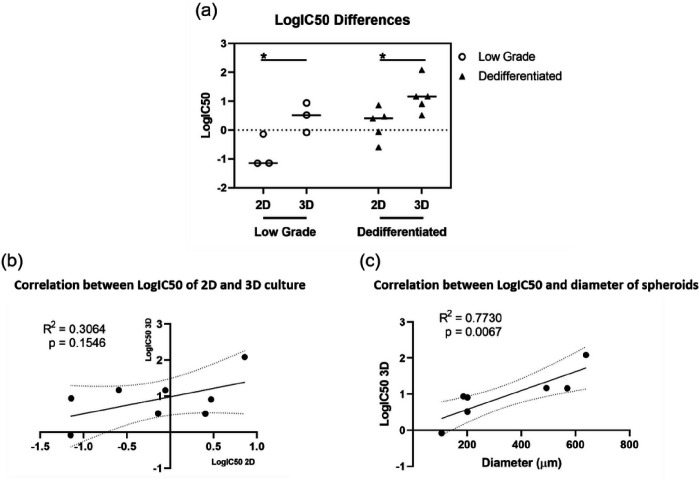
LogIC50 was related to several factors. (a) LogIC50 of four groups of cultures was plotted. Data was compared using student t-test, *p < 0.05. (b) Correlation between 2D and corresponding 3D cultures was fitted by linear regression. (c) Correlation between LogIC50 of spheroids and their sizes was fitted by linear regression.

The mean value of LogIC50 of low grade monolayer cells were − 0.81 ± 0.47, and mean value of dedifferentiated monolayer cells were 0.22 ± 0.50. the significant difference was observed between these two group of 2D cells that dedifferentiated monolayer cells were more resistant to doxorubicin treatment (p = 0.047). When comparing differences between two groups of spheroids, dedifferentiated spheroids had a higher mean value of LogIC50 at 1.17 ± 0.52, and that of low grade spheroids was at 0.46 ± 0.42, however, the difference was not significant (p = 0.13). We then compared monolayer cultures to spheroids, in both group, a significant difference (p _(low grade)_ = 0.047; p _(dedifferentiated)_ = 0.030) was found, and spheroids were more resistant to doxorubicin chemotherapy (Fig. 8a). We then fitted the curve by linear regression The trend was observed that those monolayer cells with higher resistance tended to be less sensitive to doxorubicin when they were cultured as spheroids and there was a weak correlation with R-square of 0.306 and p value of 0.155. (Fig. 8b). There was a strong correlation that those spheroids, which had a larger dimeter when treated with doxorubicin, were more resistant to the treatment. We performed the linear regression and got an R-square of 0.77, and the p value of 0.0067, which indicated the resistance was strongly related to the size (Fig. 8c)

## Discussion

In this study, we successfully generated spheroids not only from HT1080 and SW1353 cell lines, but also from 8 different patient derived primary cells. The results demonstrated the feasibility of using ULA plates for 3D spheroids culture of primary CSA cells. Besides, most of the formed spheroids can be maintained more than 10 days, then cell debris around the spheroids could be observed which indicated necrosis happened. However, there was still study indicating spheroids could be maintained for a longer time ^14,16^. This might be related to cell types. By using the ultra-low attachment plates, we were able to grow spheroids as uniformed shapes and sizes, which were essential for repeatability of further studies, i.e. histology, RNA sequencing, cytotoxicity assay, etc.

We divided cells into two groups based on their original pathology, low grade CSA and dedifferentiated CSA. Two types of growth patterns were observed, which were the contraction patterns and the expansion patterns. The phenomenon was also reported by others ^17^. All low grade spheroids shrunk during culture, while some of the dedifferentiated spheroids shrunk, and the other expanded. More interestingly, S494 is the recurrence of S166, however, the S494 spheroids expanded in size from day 3 to day 10, while S166 spheroids presented the shrunken pattern over time. The possible explanation were: 1) the growth pattern was decided by proliferating ability of the cells. The cells from low grade chondrosarcoma had lower proliferation rate and invasion ability, leading to the contraction of spheroids. This could also explain the different patterns of S166 and S494 spheroids, as recurrence was usually more malignant than the original counterpart. 2) the cells originated from low grade cartilaginous component of dedifferentiated CSA tended to grow in a contraction pattern, and those from dedifferentiated component tended to expand. This was reasonable, because for each dedifferentiated CSA, both components were presented in the tumor, and the behaviors of these two components were different ^18,19^. When we dissociated the tumor, we were not sure which cells were included or dominant in spheroids. Some studies also claimed that the growth pattern of spheroids were regulated by β1 integrin ^20^ and E-cadherin ^21,22^. The β1 integrin was important for cartilage development and mediated cell-matrix interaction, which diminished cell migration in low grade CSA ^23,24^. Therefore, this might result in contraction pattern of low grade CSA spheroids. For E-cadherin, it was only expressed in certain kinds of soft tissue sarcomas but not chondrosarcoma ^25^.

Then, we mainly made comparisons of histology among monolayer cultures, spheroids and their original tumor samples. In both low grade and dedifferentiated groups, spheroids recapitulated tumor morphology better than monolayer cultures did in aspects of cellularity, ECM and cell distribution, because spheroids demonstrated higher ECM formation and more specific spatial distribution, compared with monolayers. However, in some low grade CSA groups, although spheroids were making ECM, the staining of the matrix was not the same as that of the hyaline matrix that existed in tumors. In our humble opinion, spheroids were not producing the specialized hyaline matrix, but some more fundamental component, e.g. collagen II ^14,15^.

In the cytotoxicity assay, we demonstrated that in all primary samples, except S166, spheroids were highly resistant to doxorubicin treatment, compared with corresponding monolayer cultures. This was also reported in a study of CSA 3D cell pellets, which were similar to spheroids ^26^. This indicated we might obtain false positive results if we only use monolayer cultures as models in cytotoxicity assay, which may partially explain why lots of clinical trials turned out to be disappointing from bench-side to bed-side. The chemoresistance of spheroids were related to their size that larger spheroids were more resistant to doxorubicin. In our opinion, the larger size meant greater difficulty for drug molecules to penetrate the spheroids and this was also observed in another study ^27^. Besides, when spheroids expanded, cells in the core area would get less oxygen and nutrients, as was reported that hypoxia is also a factor contributing to chemoresistance ^28^. Although the sensitivity to doxorubicin differed from one sample to another, dedifferentiated samples were relatively more resistance to doxorubicin in both forms of monolayers and spheroids compared to low grade counterparts, and this demonstrated spheroids could retain the characteristics of their original tumors in aspect of chemoresistance, as dedifferentiated CSA was generally more resistant to chemotherapies than low grade CSA ^29^.

this study was the first to compare differences between low grade CSA and dedifferentiated CSA spheroids. And it was also the first to reveal the differences and similarities among monolayer cultures, spheroids and their original tumors in aspect of histology. There were some limitations of this study: 1) due to the low incidence of CSA, we were not able to get a larger sample sizes during this study. However, we would continue collecting samples and performing assays on a larger scale in the future. 2) the histology part was semi-quantitative and it was inevitable for us to evaluate the spheroid models subjectively. To minimize the impact of personal subjective will, the evaluation of histology features, i.e. cellularity, matrix formation, cell morphology, etc., was conducted by a pathologist from UPMC Shadyside Hospital, who was not familiar with the aim of the project, and slides were also blinded before being sent for scoring. 3) we did not furtherly investigate molecular mechanisms under our findings, so in the future, we would focus on mechanism studies and try to explore more aspects of patient derived CSA spheroids, for instance, performing the bulk RNA-sequencing of all our samples to understand the relationship between our models and original tumors in genetic and molecular aspects.

## Conclusions

In conclusion, both patient derived low grade and dedifferentiated CSA spheroids recapitulated their own tumor characteristics in aspect of morphology and chemoresistance. Spheroids were novel and ideal models for laboratory research for different types of CSA.

## Figures and Tables

**Table 1:** Summary of nonlinear regression

Cell	LogIC50 2D	R squared 2D	LogIC50 3D	R squared 3D	P value
CS23	0.4715	0.9517	0.908	0.9454	< 0.0001
S564	−0.1393	0.8323	0.5185	0.9131	< 0.0001
S494	−0.5941	0.9909	1.168	0.9539	< 0.0001
S166	0.406	0.9224	0.5128	0.8574	0.3697
CS21b	0.8611	0.9639	2.086	0.8013	< 0.0001
CS15	−1.143	0.8848	0.9393	0.9039	< 0.0001
SW1353	−1.15	0.9814	−0.08262	0.9135	< 0.0001
HT1080	−0.05575	0.8937	1.163	0.6596	< 0.0001

## Data Availability

All data generated or analysed during this study are included in this published article
